# Non-parametric quantile regression-based modelling of additive effects to solar irradiation in Southern Africa

**DOI:** 10.1038/s41598-024-59751-8

**Published:** 2024-04-22

**Authors:** Amon Masache, Daniel Maposa, Precious Mdlongwa, Caston Sigauke

**Affiliations:** 1 Department of Statistics and Operations Research, National University of Science and Technology, Ascot, P.O. Box AC 939, Bulawayo, Zimbabwe; 2 Department of Statistics and Operations Research, University of Limpopo, Private Bag X1106, Polokwane, Sovenga 0727 South Africa; 3 Department of Mathematical and Computational Sciences, University of Venda, Venda Thohoyandou, 0950 South Africa

**Keywords:** Additive effects, Additive models, Non-parametric quantile regression, Pinball loss, Quantile splines, Solar irradiation, Climate sciences, Energy science and technology, Mathematics and computing

## Abstract

Modelling of solar irradiation is paramount to renewable energy management. This warrants the inclusion of additive effects to predict solar irradiation. Modelling of additive effects to solar irradiation can improve the forecasting accuracy of prediction frameworks. To help develop the frameworks, this current study modelled the additive effects using non-parametric quantile regression (QR). The approach applies quantile splines to approximate non-parametric components when finding the best relationships between covariates and the response variable. However, some additive effects are perceived as linear. Thus, the study included the partial linearly additive quantile regression model (PLAQR) in the quest to find how best the additive effects can be modelled. As a result, a comparative investigation on the forecasting performances of the PLAQR, an additive quantile regression (AQR) model and the new quantile generalised additive model (QGAM) using out-of-sample and probabilistic forecasting metric evaluations was done. Forecasted density plots, Murphy diagrams and results from the Diebold–Mariano (DM) hypothesis test were also analysed. The density plot, the curves on the Murphy diagram and most metric scores computed for the QGAM were slightly better than for the PLAQR and AQR models. That is, even though the DM test indicates that the PLAQR and AQR models are less accurate than the QGAM, we could not conclude an outright greater forecasting performance of the QGAM than the PLAQR or AQR models. However, in situations of probabilistic forecasting metric preferences, each model can be prioritised to be applied to the metric where it performed slightly the best. The three models performed differently in different locations, but the location was not a significant factor in their performances. In contrast, forecasting horizon and sample size influenced model performance differently in the three additive models. The performance variations also depended on the metric being evaluated. Therefore, the study has established the best forecasting horizons and sample sizes for the different metrics. It was finally concluded that a 20% forecasting horizon and a minimum sample size of 10000 data points are ideal when modelling additive effects of solar irradiation using non-parametric QR.

## Introduction

Literature reviews show that solar irradiation (SI) data in Southern Africa does not follow a normal distribution and sometimes contain outliers^1–4^. It is heavy-tailed to the right and platykurtic. These statistical characteristics can be attributed to the significant effects of heterogeneous meteorological features such as temperature and sunshine hours, which are characterised by rapidly fluctuating uncertainties and error distributions with infinite limits. Assuming linear effects only is an over-generalisation of SI behaviour. However, some covariates may have linear effects or even correlated, but deducing from their nature, they also have non-linear effects on SI without reasonable doubt. Thus, the structure of the relationship between SI and suspected covariates is not known. Consequently, modelling such data using parametric assumptions would not be significant and can lead to meaningless results. One of the most proper modelling approaches is non-parametric regression because here assumptions on parametric regression do not hold. Non-parametric regression is flexible, and robust and can be applied to qualitative data. Very few assumptions need to be valid and the response variable can be agnostic. After relaxing linearity assumptions, covariate effects are restricted to smooth and continuous functions. Therefore, non-parametric regression aims to have the best regression fitted function according to how the response is distributed^5^ i.e. constructing a smooth curve as a geometric representation of the effects of the covariates on the response. A wide range of non-parametric approaches have been proposed to describe SI data. Still, the application of quantile regression (QR) has been found to outperform other methods in Southern Africa. Non-parametric estimation of families of conditional quantile functions models the full distribution of the response through conditional quantiles. Koenker^6^ stipulated that quantile functional families expose systematic differences in dispersion, tail behaviour and other features concerning the covariates. QR generates the whole conditional distribution of all predicted values. Thus, a complete picture of how covariates affect the response at different quantile levels can be described. That is, QR is more generalised than conditional mean modelling^7^. Potentially different solutions at distinct quantiles can be interpreted as differences in the response to changes in covariates at various points in the conditional distribution. QR allows a more realistic interpretation of the sparsity of the covariates effects and it is naturally robust to outlier contamination associated with heavy-tailed errors^8^. However, in multivariate cases, QR lacks a description of the additive effects of the covariates. Instead, non-parametric QR additive models have been found to handle the curse of dimensionality quite well while retaining great flexibility^9^. Such additive models are flexible regression tools that manipulate linear as well as non-linear effects at the same time^10^. Reference^11^ claimed that additive models provide programmatic approaches for nonparametric regression by restricting nonlinear covariate effects to be composed of low-dimensional additive pieces. The additive terms can be fixed, random or smooth effects. The modelling framework can be an application of non-parametric QR on additive effects or applying additive terms to non-parametric QR. The already existing modelling of SI lacks the application of non-parametric QR on additive effects to SI. Non-parametric quantile regression-based regression provides an attractive framework for parametric as well as nonparametric modelling of additive effects to the response characteristics beyond the conditional mean. The modelling of additive effects to SI using non-parametric QR may be better than the already existing additive modelling frameworks. Therefore, this current study explored non-parametric QR modelling frameworks when investigating additive effects on SI in Southern Africa.

### Review of related literature

The earliest study according to the best of our knowledge to apply QR when modelling SI data from Southern Africa was done by^12^. They proposed a partial linearly additive quantile regression (PLAQR) to model data from the Tellerie radiometric station in South Africa. The modelling framework consists of a parametric linear component and a non-parametric additive component. This modelling structure may work effectively because some covariates are perceived to have linear effects on SI. The PLAQR model with pairwise hierarchical interactions outperformed both support vector regression (SVR) and stochastic gradient boosting models. We concur with the authors on the idea of including pairwise interaction effects because, in our yet-to-be-published paper, we discovered that a significant number of SI data sets from Southern Africa had covariates with significant multicollinearity. Although^2^ did not apply QR in their study, their results also confirm that modelling SI with pairwise interactions included significantly improved forecasting model performances. Forecasts were further improved by extending the application of QR to combine forecasts through quantile regression averaging. Ranganai and Sigauke^13^ modelled SI data from Cape Town, Pretoria and Ritchersveld used an additive quantile regression (AQR) model as a benchmark against three other SARIMA models. AQR modelling framework is an application of the additive modelling concept on QR introduced by^14^. Though SARIMA models are known to capture seasonal variations in any data more than any modelling framework, they were often outperformed by AQR on the metrics considered. The study demonstrated that whenever covariates to SI can be accessed then QR modelling is recommended because residual modelling is inferior. However, the authors recommended the application of the SARIMA models in cases of non-existent or scanty covariates. A separate study^15^ demonstrated that AQR is also superior to extreme models in estimating extreme quantiles of SI data from Venda in South Africa except on the $\tau = 0.9999$ quantile level. This shows that additive non-parametric QR is a very powerful modelling framework when forecasting the whole response distribution, and cyclical and seasonal variations in SI. A quantile generalised additive model (QGAM) is a new approach that was introduced by^16^ where smooth effects estimated by a generalised additive model (GAM) are taken as inputs to a QR model. That is, performing QR on smooth function outputs from a GAM. The modelling framework is still very new in such a way that its literature is very limited. Among studies in Africa, we can only cite^17^ who modelled the additive effects of fertility rate and birth rate on human live births. The QGAM was found to be a robust alternative to a GAM on most quantile levels although they had the same adjusted R-sqaure at the 50th quantile level. Recently,^18^ studied spatially compounding climate extremes using QGAMs and they could predict the extremes more accurately than the conventional peak-over-threshold models. However, the outperformance was discovered in some regions, while it was inferior in other regions. This means that we can perceive that among other forecasting frameworks, QGAMs likewise perform differently in different geographical locations. QGAMs have not been used to forecast SI anywhere else except as a means of combining forecasts done by^1^, according to the best of our knowledge. However, the approach was inferior to other forecasts combining frameworks. As a result, it is not a good forecast combination method. We argue that the QGAM framework is better applied as a forecast-generating model rather than a forecast combination. It is a novel additive effect modelling in climate science applications and presents key advantages over residual modelling. QGAMs remove the need for direct identification and parameterisation since they model all quantiles of the distribution of interest. Thus, making use of all information available does not require any prior information about the relationships between the response variable and its covariates. Therefore, we propose to compare the predictive performance of QGAM against PLAQR and AQR using SI data from Southern Africa.

### Contributions and research highlights

SI data is known to be skewed and plakurtic, and assumptions on parametric modelling do not hold well. As a result, non-parametric quantile regression, where normality assumptions are ignored can best model SI. Therefore, to the already existing work on QR modelling of SI data in Southern Africa, the main contribution of this study is to introduce the idea of predicting SI using a QGAM. In this modelling framework, a QR approach was applied to a generalised additive model. That is, hybridising a GAM with a QR model. This non-parametric modelling framework is new to SI data. Non-parametric QR-based models namely PLAQR and AQR have been used before to model SI in independent separate studies. However, they have not been compared in their forecasting performances. Although PLAQR and AQR were best in those separate studies, they have their weaknesses. As a result, the other contribution of this study is the comparison of predictive performances of the three non-parametric quantile regression-based models on different geographical locations. We perceive that probabilistic forecasting can be affected by the spatial distribution of data sources. Grid differences, location elevation, climatic conditions and their combinations may affect forecasting models. The last contribution of this study is to investigate separately how forecasting horizon and sample size affect the performance of the additive models. This helps identify the forecasting horizon up to which the QR-based models retain their predictive performances. It is generally perceived that the more data points we have the more a training model is effective. This is because more data points give more information to train. As a result, a supervised machine learning model like non-parametric QR-based can learn more about the data given. However, the question is, if the sample size is increased continuously then do QR-based models also continuously increase their performances? That is, we also established the smallest sample size that can be considered when training a non-parametric QR-based model.

In this research study, we applied Lasso via hierarchical interactions to select significant covariates and interaction effects from each location. We considered covariates recommended from our study that is still under review. PLAQR, AQR and QGAM models were trained on each set of locational selected covariates at all quantile levels. The residual mean square error (RMSE) validation metric was used to find the best quantile level for the three models. The best quantile level was then used for comparison investigations on the three models. Breusch–Godfrey and Box–Ljung tests were used to check on the assumption of residual serial autocorrelation. We also validated the models using the R-square as well as cross-validation correlations to check whether the models were overfitting the data or not. The accuracy of the additive models was compared using the mean absolute scaled error (MASE). MASE is one of the most appropriate accuracy metrics when the response has zero or near zero values. Since the main objective of QR is to minimise the pinball loss, then it became the priority performance evaluation metric in this study. Other probabilistic forecasting performance evaluation metrics namely the Winkler score, Coverage Probability (CP) and Continuous Rank Probability Score (CRPS) were used to compare the predictive performances of the models. The QGAM outperformed both the PLAQR and AQR models in most scenarios of forecasting performance evaluations. However, it was not superior at all when using the Winkler score. The performance evaluations were also done in different locations, increasing forecasting horizons and increasing sample sizes.

The study helps develop SI modelling frameworks that can be used to accurately forecast solar power. Accurate forecasts of solar power improve the stability of solar power generation and effective management of renewable resources. Exploration of multisite modelling captures variations in weather conditions in the region and allows the evaluation of data management systems at different ground-based radiometric stations. Evaluation of forecasting horizons and sample sizes helps inform the body of knowledge and the solar power generation industry of the forecasting horizons thresholds and minimum sample sizes to be considered when predicting solar irradiation.

## Methodology

### Non-parametric quantile regression concept

The $\tau$th quantile is the minimiser of the expected loss $\rho _{\tau }$ with respect to $Q_{Y_i}(\tau |x_i)$, where by definition

1 $$\begin{aligned} Q_{Y_i}(\tau |x_i)=\text{ F}^{-1}_{Y_i}(\tau |x_i)\equiv f_i(x_i,\tau ), \end{aligned}$$

and F is the conditional cumulative distribution function (CDF) of *Y*. When approximating the quantile loss function (where *y* is the observation used for forecast evaluation and $\tau _q$ is the *q*th quantile for $q= 1,2,..., 99$) we obtain the quantile estimator

2 $$\begin{aligned} {\hat{Q}}_{Y_i}(\tau |x_i)=x^T{\hat{\beta }}=g(x_i,{\hat{\beta }}(\tau )), \end{aligned}$$

where $$g=\text{ inf }\left\{ y: \text{ F }(y|x)\ge \tau \right\} $$,

3 $$\begin{aligned} {\hat{\beta }}(\tau )=\underbrace{{\text{ argmin }}}_{b \in \beta }\sum ^n_{i=1}\rho _{\tau }(y_i-g(x_i,\beta ))~~\text{ and }~~\tau \in T,~T=[\varepsilon ,1-\varepsilon ]~\text{ for } \text{ some }~~0<\varepsilon <1. \end{aligned}$$

$\text{ F}_i$ should be continuous with continuous density $f_i(\tau )=g(x_i,\beta (\tau ))$ uniformly bounded away from 0 and $\infty$ at some points as a first regularity condition to the minimisation problem in Eq. (3). To ensure that the objective function of the problem has a unique minimum at $\beta$ and is sufficiently smooth we consider the following assumptions from^11^.

- there exist positive constants $a_0$ and $a_1$ such that,
  4 $$\begin{aligned} a_0||\beta _1-\beta _2||\le \left( \frac{1}{n}\sum ^n_{i=1}(g(x_i,\beta _1)-g(x_i,\beta _2))^2\right) ^{\frac{1}{2}}\le a_1||\beta _1-\beta _2||,~~\text{ for }~~\beta _1,\beta _2 \in \beta , \end{aligned}$$
- and there also exist positive definite matrices $\text{ M}_0$ and $\text{ M}_1(\tau )$ such that:
  1. $$\underbrace{{\text{ lim }}}_{n\rightarrow \infty }\frac{1}{n}\sum ^n_{i=1}{\dot{g}}_i{\dot{g}}^{T}_i=\text{ M}_0$$,
  2. $$\underbrace{{\text{ lim }}}_{n\rightarrow \infty }\frac{1}{n}\sum ^n_{i=1} f_i{\dot{g}}_i{\dot{g}}^{T}_i=\text{ M}_1(\tau )$$, and
  3. $$\underbrace{{\text{ max }}}_{i=1,2, \ldots ,n} \frac{||{\dot{g}}_i||}{\sqrt{n}}\rightarrow 0, $$
  where $${\dot{g}}=\frac{\partial g(x_i,\beta )}{\partial \beta }|_{\beta =\beta _0}.$$

A provision of uniform linear representation and convergence of the minimisation process is given by the following theorem.

#### Theorem 1

*Under the above assumptions* ^6^

5 $$\begin{aligned} \sqrt{n}\left( {\hat{\beta }}_n(\tau )-\beta (\tau )\right) \sim N\left( 0, \tau (1-\tau )\text{ M}^{-1}_1\text{ M}_0\text{ M}^{-1}_1\right) . \end{aligned}$$

*The minimiser of the problem in Eq.* (3) *by choice of a tuning parameter (or a penalty) satisfies the following*:

i. *The number of terms*, $n_-$, *with* $y_i<g(x_i,\beta )$ *is bounded above by* $\tau n$.
ii. *The number of terms*, $n_+$, *with* $y_i>g(x_i,\beta )$ *is bounded above by* $(1-\tau ) n$.
iii. *For* $n\rightarrow \infty$, *the fraction* $\frac{n_-}{n}$ *converges to* $\tau$ *if* Pr(*y*|*x*) *is completely continuous*.

But Pr(*y*|*x*) is not known, so it has been suggested by^19^ to resort to minimising the regularised empirical risk

6 $$\begin{aligned} R_{reg}(f):=\frac{1}{n}\sum ^n_{i=1}\rho _{\tau }(y_i-f(x_i))+\frac{\lambda }{2}||h||^2_H,~~~\text{ for }~f=h+a,~a\in R~\text{ and } \text{ regularised }, \end{aligned}$$

where $R(f)=E_{\text{ Pr }(y|x)}[\rho _{\tau }(y-f(x))]$ is the empirical risk and $||.||_H$ is the reproducing Kernel Hilbert space (RKHS) norm.

#### Lemma 1

*The minimiser of Eq*. (6) *when assuming that* *f contains an unregularised scalar term satisfies*:

i. *The number of terms*, $n_-$, *with* $y_i<f(x_i)$ *is bounded above by* $\tau n$.
ii. *The number of terms*, $n_+$, *with* $y_i>f(x_i)$ *is bounded above by* $(1-\tau ) n$.
iii. *If* (*x*, *y*) *is drawn iid from a continuous distribution* Pr(*y*|*x*) *and the expectation of the modulus of absolute continuity of its density satisfying the limit of* $E[\epsilon (\delta )]$ *as* $\delta \rightarrow 0$ *is equal to zero with probability one, then* $\frac{n_-}{n}$ *converges to* $\tau$ *asymptotically*.

### Quantile splines

Now, the quantile function in Eq. (1) can be more generalised as

7 $$\begin{aligned} Q_Y(\tau |X)=g(X^T_1\beta _1,X^T_2\beta _2, \ldots ,X^T_m\beta _m), \end{aligned}$$

where *m* is much smaller than the covariate space dimension. The minimisation problem in Eq. (3) may involve additive models of the form

8 $$\begin{aligned} Q_Y(\tau |X)=\mu _{\tau }+\sum ^n_{i=1}g_i(x^T_i,\beta _i)+e_{\tau }, \end{aligned}$$

where $\mu _{\tau }$ is an unknown constant and $g_i$ is an additive term which is a function of a smooth function. We assume the quantile error term, $e_{\tau }$ to be uncorrelated to include linear effects in all of the models when estimating the generalised quantile function. The additive form has easy interpretability and visualisation. Quite several local polynomial methods have been developed for estimating the additive models, but do not work well for QR applications. Instead, quantile smoothing has been traditionally done competitively between kernel and spline functions to model the non- linear effects. However, multicultural tendencies have weakened the competition with consideration of the two through penalty methods. Penalised quantile smoothing splines have been found to avoid the arbitrary choice of the number and positions of knots. That is, the non-parametric conditional quantile functions can now be estimated by solving the following problem:

9 $$\begin{aligned} \text{ min}_{g\in \textbf{S}}\sum ^n_{i=1}\rho _{\tau }(y_i-g_i(x^T_i,\beta _i))+\lambda P(g), \end{aligned}$$

where $\textbf{S}$ is a Sobolev space of real-valued functions, $x_i=(x_{i1},x_{i2}, \ldots ,x_{id},)$ is an element of *d* dimensional space of real numbers and *P* is the penalty term designed to control the roughness of the fitted function, ${\hat{g}}$.

Now, any solution ${\hat{g}}$ must interpolate itself at the observed $\left\{ x_i \right\}$ i.e. we have to find the smoothest interpolant of the points $\left\{ (x_i,y_i),~i=1,2, \ldots ,n \right\}$ in the sense of solving

10 $$\begin{aligned} \text{ inf }\left\{ ||g^{(d)}||_p:~g\in \textbf{S}^d_p,g(x_i)=y_i,~i=1,2, \ldots ,n \right\} \end{aligned}$$

and the functions for which the infima are attained.

Let $z_1,z_2, \ldots ,z_N~ (z_i \ne z_{i+1}, ~i=1,2, \ldots ,N-1)$ be given real fixed data, then for each $t\in T_N$ set

$$\begin{aligned} \textbf{S}^d_p(t,z)=\left\{ g:g\in \textbf{S}^d_p, ~g(t_i)=z_i,~i=1,2, \ldots ,N \right\} , \end{aligned}$$

where $T_N=\left\{ t:t=(t_1,t_2, \ldots ,t_N),~0\le t_1\le t_2\le \ldots \le t_N \right\}$ and $p\in (1,\infty )$. Thus solving

11 $$\begin{aligned} \underbrace{\text{ inf }}_{t \in T_N}~ \text{ inf }\left\{ ||g^{(d)}||_p:~g\in \textbf{S}^d_p(t,z),g(x_i)=y_i,~i=1,2, \ldots ,n \right\} . \end{aligned}$$

$\textbf{S}^d_p$ is the Sobolev space of real-valued functions with $d-1$ absolutely continuous derivatives of which the *d*th derivative exist as a function in $L_p[0,1]$ which means that

12 $$\begin{aligned} \textbf{S}^d_p=\left\{ g:g(x)=\sum ^{d-1}_{i=0}a_ix_i+\frac{1}{(d-1)!}\int ^1_0 (x-y)^{h-1}_+h(y)dy,~h\in L_p,a\in R \right\} , \end{aligned}$$

where $a_i=\frac{g^{(d)}(0)}{i!},~i=0,1, \ldots ,d-1$ and $h \equiv g^{(d)}\in L_p$. If we assume the following facts;

- $$(z_i-z_{i-1})(z_{i+1}-z_i)<0,~i=1,2, \ldots ,N,$$
- $$N>d$$ and
- $$t_1=0$$ and $$t_N=1$$,

then there exists a solution to the problem (11) $g\in \textbf{S}^d_p$ which must be of a particular form and oscillate strictly between $(z_i)^N_1$. This solution is a unique necessary and sufficient solution to problem (9).

Now, it means that solving the problem (10) is equivalent to solving

13 $$\begin{aligned} \text{ inf }\left\{ ||h||_p:~\int ^1_0 B_{i,d}(y)h(y)dy=E_i,~i=1,2, \ldots ,N-d \right\} , \end{aligned}$$

which can be shown that

14 $$\begin{aligned} h(y)=\left| \sum ^{N-d}_{i=1}\beta _i B_{i,d}(y) \right| ^{q-1} \text{ sign } \left( \sum ^{N-d}_{i=1}\beta _i B_{i,d}(y) \right) , \end{aligned}$$

is the unique solution to the problem, where $$ B_{i,d}$$ is a positive multiple of a B-spline of degree $$d-1$$ with knots $$t_i,t_{i+1}, \ldots ,t_{i+d}$$. $$E_i=g[t_i,t_{i+1}, \ldots ,t_{i+d}]$$ is obtained by applying the *d*th divided difference at the points $$t_i,t_{i+1}, \ldots ,t_{i+d}$$ to $$g\in \textbf{S}^d_p(t,z)$$. This follows that

15 $$\begin{aligned} g_p(x)=\sum ^{d-1}_{i=0}a_ix_i+\frac{1}{(d-1)!}\int ^1_0 (x-y)^{h-1}_+h(y)dy \end{aligned}$$

is a unique solution to the problem (10) when $(a_i)^{d-1}_0$ is uniquely determined so that $g_p(t_i)=z_i,~i=1,2, \ldots ,d$. Therefore,

16 $$\begin{aligned} g_p\in \textbf{S}^d_p(t,z). \end{aligned}$$

Now,^20^ expanded the original space of real functions to

17 $$\begin{aligned} W^2=\left\{ g:g(x)=a_0+a_1x+\int ^1_0(x-y)_+du(y),~a_i\in R, i=0,1 \right\} \end{aligned}$$

and replaced the $L_1$ penalty on the smooth effects with a total variation penalty on $g'$ defined as $V(g')=\int |g''(x)|dx$ to have the following theorem.

#### Theorem 2

*The function* $g\in W^2$ minimising

18 $$\begin{aligned} \sum \rho _{\tau }\left\{ y_i-g(x_i)\right\} +\lambda V(g') \end{aligned}$$

*is a linear spline with knots at the points* $x_i,~i=1,2, \ldots ,d$.

Therefore, we can deduce that $g(x)=\sum ^n_{j=1}s_j(x)$ and the $s_j's$ are the additive smooth effects. The smooth effects are defined in terms of spline basis as follows;

19 $$\begin{aligned} s_j(x)=\sum ^K_{k=1}\beta _{jk}B_{jk}(x_j). \end{aligned}$$

#### Remark

The first derivative of $g~(g':R\rightarrow R)$ is continuous and if we denote $\nabla ^2g(x)$ as a Hessian matrix of *g* and ||.|| as a Hilbert Schmidt norm for matrices then

20 $$\begin{aligned} V(\nabla g)=\int ||\nabla ^2g(x)||dx. \end{aligned}$$

That is, $\lambda V(\nabla g)$ becomes the $L_1$ form of the roughness penalty and is a linear spline.

### Regression coefficients estimation

The estimation of regression coefficients heavily depends on how the additive effects are being modelled. When considering linear effects as well as additive effects a PLAQR model can be fitted while an AQR is fitted when considering a complete additive model. In our study, we propose fitting a QGAM which can be more efficient and accurate than an AQR.

#### Partial linearly additive quantile regression model

Notwithstanding that some of the covariates may have linear effects on SI then it is prudent to consider a non-parametric QR model that includes the linear effects. It may not be practical to assume that all covariates are non-linear. Such a model was introduced by^9^, which has a non-parametric component and an additive linear parametric component.

i.e.

21 $$\begin{aligned} y_t=\mu _{\tau }(t)+\sum ^{m_1}_{i=1}s_{it,\tau }(x_{it})+\sum ^{m_2}_{j=1}\beta _{jt,\tau }z_{jt}+e_{\tau }(t), \end{aligned}$$

where $\mu _{\tau }(t)$ is an unknown constant, $x_{it}\in \textbf{X}_{m_1 \times 1}$ are continuous variables for $i=1,2, \ldots ,m_1$, $s_{it,\tau }\in \textbf{S}$ are the smooth functions, ${z_{jt}\in \textbf{Z}}_{m_2 \times 1}$ are the linear covariates for $j=1,2, \ldots , m_2$ and $e_{\tau }$ is the quantile error term such that

$$\begin{aligned} 0< \text{ Pr }(e \le Y|\textbf{XZ})=\tau (\text{ a.s.})<1. \end{aligned}$$

If we assume that $\textbf{X}$ takes values in $\chi \equiv [-1,1]^{m_1}$ and letting

$$\begin{aligned} \mathbf{S_{\tau }}(\textbf{X})=\mu _{\tau }(t)+\sum ^{m_1}_{i=1}s_{it,\tau }(x_{it}), \end{aligned}$$

then we can write the PLAQR model in matrix notation as follows.

22 $$\begin{aligned} \textbf{Y}=\mathbf{S_{\tau }}(\textbf{X})+\textbf{Z}^T\mathbf{\beta _{\tau }}+e_\tau , \end{aligned}$$

where $\textbf{X}=(x_{1t},(x_{2t}, \ldots ,x_{m_1t},)\in \chi .$ If we also let $\lambda _i$ be a non-negative penalty then the quantile estimates of the PLAQR model can be found by minimising

23 $$\begin{aligned} \sum ^n_{i=1}\rho _{\tau }\left( Y_i-Z^T_i\beta -s_{\tau }(X_i)\right) +\sum ^n_{i=1}\lambda _i \int (s''(t))^2dt, \end{aligned}$$

where the $\rho _{\tau }(u)=u(\tau -I(u<0))$ is the pinball loss function.

#### Additive quantile regression model

The AQR model proposed by^14^ and algorithm further developed by^16^ gives flexibility when modelling non-linear effects beyond the conditional mean. The non-parametric components are composed of low-dimensional additive quantile pieces. Thus, an application of additive modelling on QR. As a result, the Laplacean quantile fidelity replaces the Gaussian likelihood in conditional mean regression. $L_1$-norms replace $L_2$-norms as measures of roughness on fitted functions. A generic AQR model for non-linear and varying regression coefficient terms can be written as an extension of a linear predictor with a sum of nonlinear functions of continuous covariates^14^ as follows:

24 $$\begin{aligned} Q_{y_i}(\tau )=x^T_i\beta _0+\sum ^J_{j=1}g_j(x_{ij})+e_{\tau }. \end{aligned}$$

Now, the following form of problem (9) is solved to estimate the continuous functions *g* and regression coefficients;

25 $$\begin{aligned} \underbrace{\text{ arg } \text{ min }}_{\beta ,g}\sum ^n_{i=1} \rho _{\tau }\left\{ y_i-x_i^T\beta _0-\sum ^J_{j=1} g_i(x_{ij})\right\} + \lambda _0||\beta _0||_1+\sum ^J_{j=1}\lambda _j V(\nabla g_j), \end{aligned}$$

where the pinball loss function is defined as in PLAQR model fitting. Though the model can be estimated by linear programming algorithms as in linear QR, penalty methods are applied because the known selected basis functions can be included in the design matrices^14^. As a result, sparse algebra is the supplant basis expansion through either performance-oriented iteration for large data sets (PIRLS) or the Newton algorithm.

#### Quantile generalised additive model

Additive effects of the covariates are modelled by considering the smooth effects estimated by a GAM as inputs to a linear QR. That is, a conditional quantile is modelled as a sum of unknown smooth functions^18^. Fasiolo^21^developed a regression coefficient estimation process by introducing a learning rate $\frac{1}{\sigma }>0$ and positive semidefinite matrices **M** to a penalised pinball loss as follows:

26 $$\begin{aligned} {\hat{\beta }}_{\tau }\in \underbrace{\text{ arg } \text{ min }}_{\beta \in R^d}\sum ^n_{i=1}\frac{1}{\sigma } \rho _{\tau } \left\{ y_i-g_i(x^T_i,\beta _i) \right\} +\frac{1}{2}\sum ^m_{j=1}\lambda _j \beta ^T \text{ M}_j\beta , \end{aligned}$$

where $\lambda _j$ are positive smoothing parameters. The learning rate determines the relative weight of the loss and penalty while the matrices penalise the wiggliness of the corresponding smoothing effect. The pinball loss function is replaced by a scaled pinball loss called the extended log-f (ELF) loss function;

27 $$\begin{aligned} \rho ^*_{\tau }(y-g)=(\tau -1)\frac{y-g}{\sigma }+\lambda \text{ log }(1+e^{\frac{y-g}{\sigma }}),~~~~\lambda >0. \end{aligned}$$

The ordinary pinball loss function is piecewise linear and has discontinuous derivatives while the ELF loss leads to more accurate quantiles because it is an optimally smoothed version. Thus, it enables efficient model fitting through the use of smooth optimisation methods. Now, the regression coefficients being the solution to problem (26) are obtained as a vector of maximum a posteriori (MAP) estimator, ${\hat{\beta }}_{\tau }$. A stable estimation can be done by exploiting orthogonal methods for solving least squares problems.

### Performance evaluations

The main model forecasting performance evaluation metrics considered in this study are the pinball loss function, Winkler score, CP and CRPS. The pinball loss measures the sharpness of a QR model. It is a special case of an asymmetric piecewise linear loss function defined as follows:

28 $$\begin{aligned} \text{ Pinball }({\hat{Q}}_{y_t}(q),y_t,q)=\left\{ \begin{array}{cc}(1-q)({\hat{Q}}_{y_t}(q)-y_t),&{}\text{ for }~~~~y_t<{\hat{Q}}_{y_t}(q)\\ ~&{}~\\ q(y_t-{\hat{Q}}_{y_t}(q)),&{}\text{ for }~~~~y_t~\ge {\hat{Q}}_{y_t}(q), \end{array} \right\} \end{aligned}$$

where ${\hat{Q}}_{y_t}(q)$ is the predicted SI at the $q^{th}$ quantile level and $y_t$ is the actual SI.

CP runs numerous samples in which a wide range of possible outcomes is generated for each sample. Then, this range of possible outcomes can be compared to the actual value to see if they properly account for it in its range. That is, if, for example, a $95\%$ prediction interval covers at least $95\%$ of the observed then the model is reliable, well-calibrated or unbiased.

The Winkler score then becomes a trade-off between coverage and the prediction interval width (PIW). It is the length of the prediction interval plus a penalty if the observation is outside the interval. It is defined as,

29 $$\begin{aligned} W_{\alpha ,t}=\left\{ \begin{array}{ll} (u_{\alpha ,t}-l_{\alpha ,t})+\frac{2}{\alpha }(l_{\alpha ,t}-y_t),&{}\text{ for }~~~y_t<l_{\alpha ,t}\\ (u_{\alpha ,t}-l_{\alpha ,t}),&{}\text{ for }~~~~ l_{\alpha ,t}~\le y_t\le u_{\alpha ,t}\\ (u_{\alpha ,t}-l_{\alpha ,t})+\frac{2}{\alpha }(y_t-l_{\alpha ,t}),&{}\text{ for }~~~y_t<u_{\alpha ,t}, \end{array}\right\} \end{aligned}$$

where $[l_{\alpha ,t},u_{\alpha ,t}]$ is the $(100-\alpha )\%$ prediction interval at time t.

We evaluated how the models predicted the whole forecast distribution (rather than particular quantiles) by obtaining a CRPS by averaging quantile scores over all values of *p*. That is,

30 $$\begin{aligned} \text{ CRPS }(\hat{F_{p}},p)=\int ^{\infty }_{-\infty }\left( \hat{F_{p}}(y)-1_{p\le y} \right) ^2dy, \end{aligned}$$

where $\hat{F_{p}}$ is the predictive cumulative density function and 1 is an indicator.

## Data analysis and results

### Data sources

**Figure 1:**
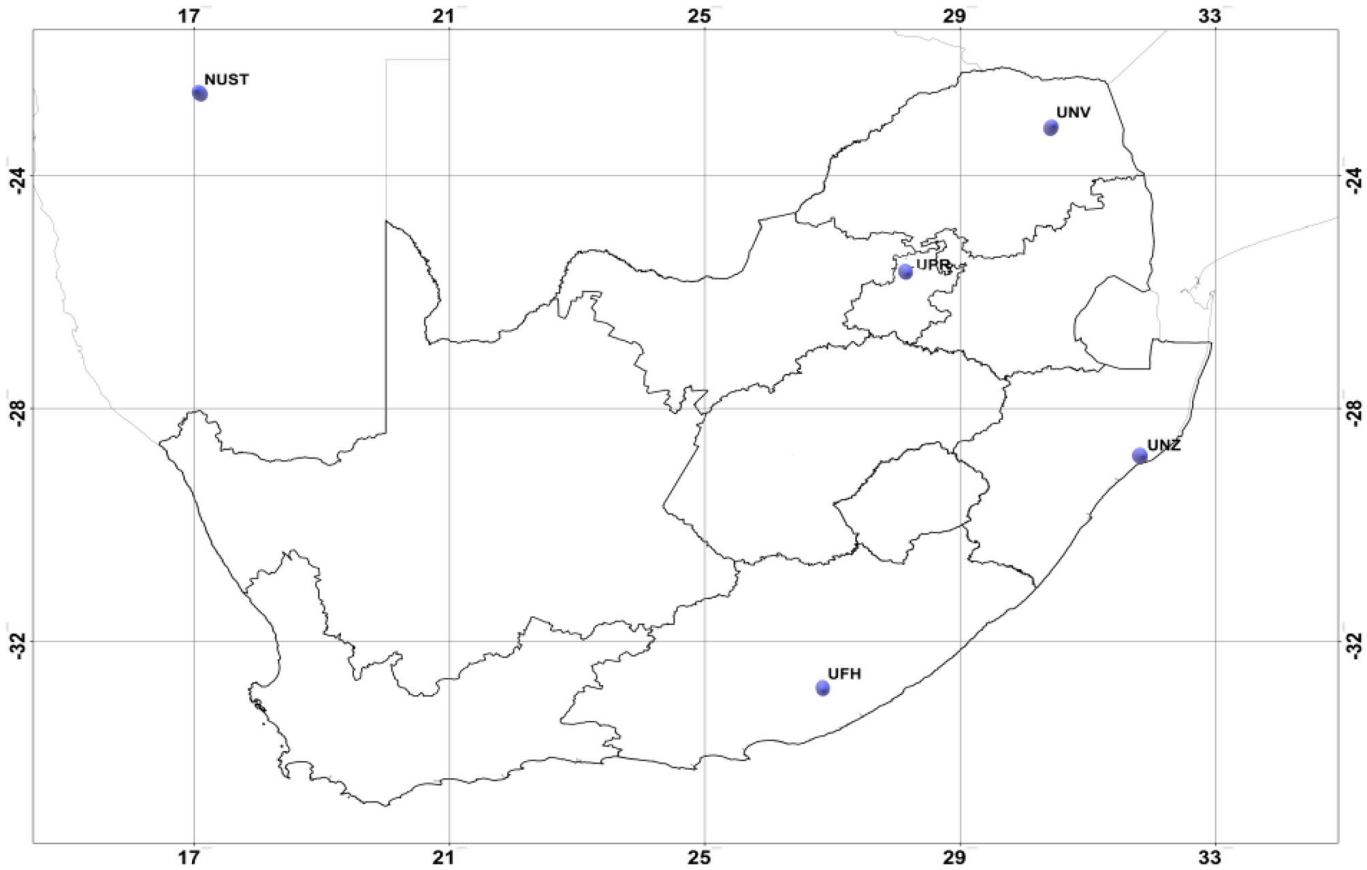
Map showing the geographic positions of the radiometric stations considered from Southern Africa: Source,^1^ Edited.

Five among several other radiometric stations considered in this study are geographically located as shown in Fig. 1 and Table 1. The stations were Namibia University of Science and Technology (NUST), University of Fort Hare (UFH), University of Kwazulu-Natal (UKZN) Howard College, University of Pretoria (UPR) and University of Venda (UNV). Data is uploaded from the stations by the Southern Africa Universities Radiometric Association Network (SAURAN) into their database and can be accessed through their website. The five stations shown in the map were the only ones that had consistent hourly data and manageable missing observations for the same period of March 2017 up to June 2019.

**Table 1:** Geographic locational description of ground radiometric stations considered.

Station	Lattitude	Longitude	Elevation	Location
NUST	− 22.56500053	17.07500076	1683 m	Windhoek
UFH	− 32.78461075	26.84519958	540 m	Alice
UKZN	− 29.87097931	30.97694969	150 m	Durban
UPR	− 25.75308037	28.22859001	1410 m	Pretoria
UNV	− 23.13100052	30.42399979	628 m	Venda

### Data exploration

#### SI distribution

**Figure 2:**
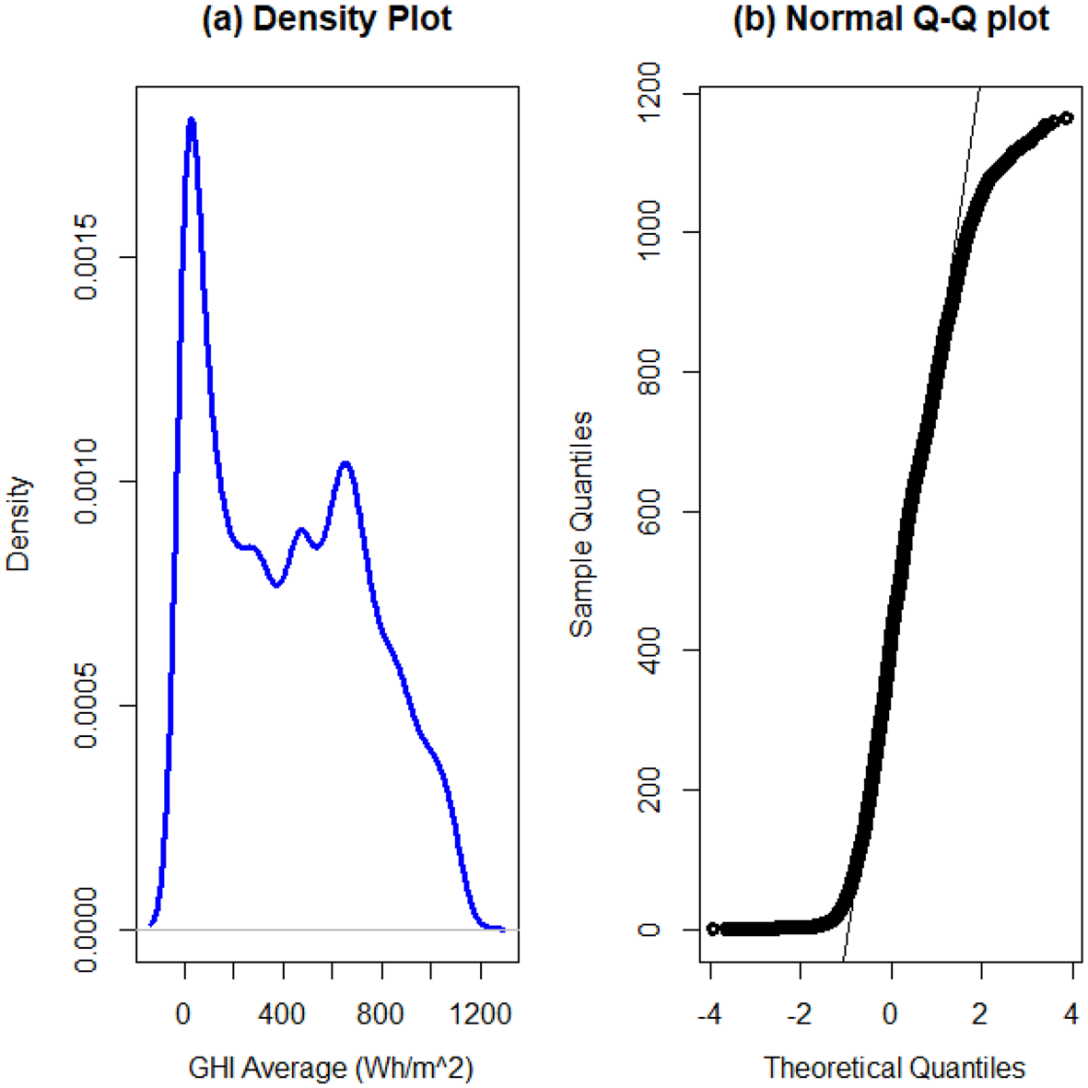
General pattern exhibited by the density and normal Q–Q plots constructed for GHI from Pretoria, Venda, Durban, Windhoek and Alice SI data sets.

**Figure 3:**
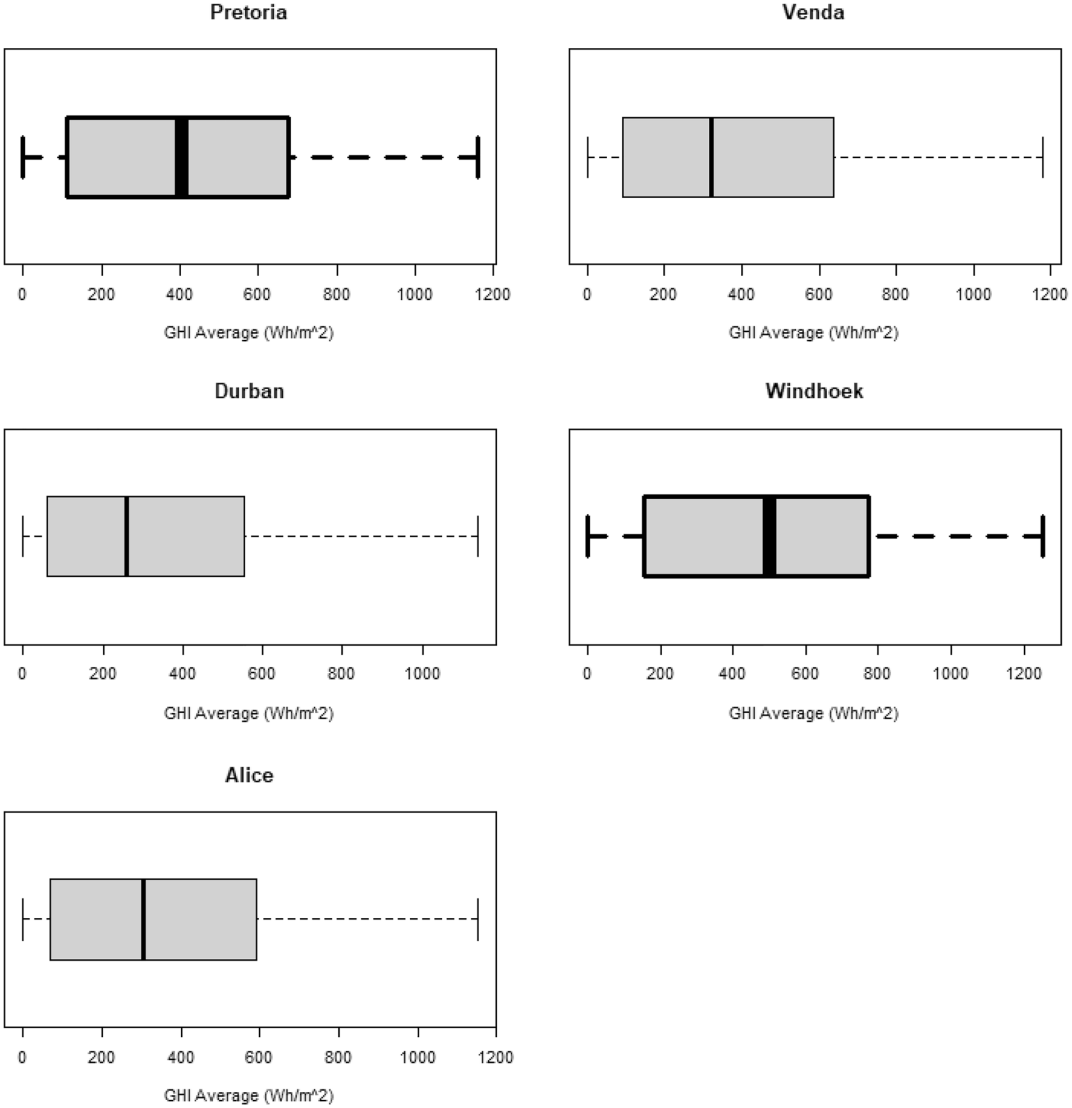
Box plots showing distributions of GHI from the five locations.

In this study, solar irradiation was measured as global horizontal irradiance (GHI). Distributions of GHI from the five locations had similar densities and Q–Q plots as those shown in Fig. 2. The distribution exhibited in Fig. 2 shows the general curve of the density plots and pattern followed by the Q–Q plots. The two plots show that GHI does not follow a normal distribution. The data exhibited asymmetric distributions in all locations as shown by box plots in Fig. 3. The box plots also show that GHI is skewed to the right-hand side and heavily tailed. A Jarque–Bera (JB) test was done on all locations to confirm the non-normality in the data. It is a goodness-of-fit test of whether sample data have the skewness and kurtosis matching a normal distribution. Among the most effective normality tests, the JB test is the most suitable test for large sample sizes. The parametric test presumes that the data originates from a particular distribution. Distributions of GHI from different locations were fitted in one of our studies^22^. Since all p-values were less than 0.05 (shown in Table 2) then the results confirmed that solar irradiation does not follow a normal distribution. The descriptive statistics in Table 2 also indicate that SI is positively skewed and platykurtic. These results are consistent with results from^22^ and several other studies.

**Table 2:** Descriptive statistics of GHI from Pretoria, Venda, Durban, Windhoek and Alice.

Location	Min	1stQu.	Median	Mean	3rdQu.	Max.	Skewness	Kurtosis	JB p-value
Pretoria	0.1002	111.175	404.583	420.464	675.482	1162.004	0.289	− 1.095	$1.98 \times 10^{-16}$
Venda	0.1002	92.150	322.355	379.684	635.695	1179.160	0.480	− 0.940	$2.09 \times 10^{-16}$
Durban	0.1010	59.995	260.800	333.450	552.175	1141.000	0.668	− 0.645	$1.89 \times 10^{-16}$
Windhoek	0.1000	157.800	501.800	490.000	772.100	1251.300	0.132	− 1.196	$2.16 \times 10^{-16}$
Alice	0.1010	67.537	301.680	361.852	583.160	1154.604	0.580	− 0.775	$1.00 \times 10^{-3}$

#### Variable selection

The following covariates; hour, temperature (Temp), relative humidity (RH), barometric pressure (BP), wind speed (WS) and wind direction (WD) were considered in this study. The descriptive statistics of the covariates are shown in Appendix A. One of the assumptions to hold valid when applying additive models to predict a response variable is that the covariates are stationary. As a result, the Kwiatkowski–Phillips–Schmidt–Shin (KPSS) test was done to check on the stationarity of the covariates. Among the most effective stationarity tests available the KPSS test is the most appropriate one for large samples. The KPSS test results in Table 3 indicate that all covariates were stationary except WD from Alice and WS from Durban (It does not matter to consider the stationarity of time, in this study time is measured in hours). On stationary covariates, the p-values were less than 0.05. That is, the null hypothesis that ‘The variable is not stationary’ was rejected and we conclude that there is enough evidence to support the assumption that the covariate is stationary. Non-stationary covariates were differenced to achieve stationarity. Lasso hierarchical pairwise interaction selections (using the ‘hierNet’ R package by^23^) with Lag1 and Lag2 of GHI included to model trend in SI time series^12^. Hour, Temperature, RH, Lag1 and Lag2 had significant effects on GHI in all locations. However, BP had a significant effect on solar irradiance in Alice only while WD had a significant effect in Alice and Durban. WS was not significant in Alice and Venda.

**Table 3:** KPSS test p-values for GHI and covariates considered from each location.

Variable	Windhoek	Alice	Durban	Pretoria	Venda
GHI	0.0344	0.0100	0.0496	0.0100	0.0598
Hour	0.0214	0.0258	0.0744	0.0362	0.0100
Temp	0.0100	0.0097	0.0099	0.0100	0.0089
RH	0.0096	0.0100	0.0100	0.0095	0.0100
WS	0.0381	–	0.0530	0.0745	–
WD	–	0.0860	0.0634	–	–
BP	0.0098	–	–	–	–
Lag1	0.0354	0.0089	0.0493	0.0100	0.0586
Lag2	0.0364	0.0100	0.0488	0.0088	0.0570

### Model validations

The best quantile level for each model was identified by comparing the root mean square error (RMSE) and $\tau =0.5$ as the best quantile level for all models fitted at all locations. As a result, all models were trained and fitted at the 50th quantile level. The proposed QGAM was fitted using the ‘mgcViz’ package developed by^24^ while the ‘plaqr‘ package by^25^ and ‘quantreg‘ by^26^ were used to fit the PLAQR and AQR models respectively. All three models were validated by checking whether the assumption of no residual serial autocorrelation was holding using the Breusch–Godfrey (BG) test and the Ljung Box test. The BG test requires the assumption of predeterminedness. The assumption was considered valid to proceed with the BG test because all covariates used were stationary. The Ljung Box requires the assumption of strict exogeneity. Since all covariates considered do not depend on solar irradiance but rather SI depends on meteorological features and the error terms of the models fitted. Both the BG test and the Ljung–Box test had p-values greater than 0.05 (Table 4) indicating that the null hypothesis, ’there is no residual serial autocorrelation’ could not be rejected. This meant that all models fitted had no serial autocorrelation of the errors.

**Table 4:** Model validation metrics on the five different trained data.

Location	Model	BG test	Box Ljung	R-square	$80\%$CV	$20\%$CV
		p-value	p-value			
Windhoek	PLAQR	0.117	0.501	0.929	0.963	0.964
	AQR	0.247	0.876	0.910	0.953	0.954
	QGAM	0.147	0.858	**0.934**	0.966	0.967
Alice	PLAQR	0.329	0.204	0.923	0.962	0.961
	AQR	0.631	0.616	0.913	0.957	0.956
	QGAM	0.631	0.709	**0.930**	0.966	0.964
Durban	PLAQR	0.998	0.664	0.920	0.957	0.959
	AQR	0.889	0.436	0.911	0.952	0.955
	QGAM	0.889	0.208	**0.924**	0.960	0.962
Pretoria	PLAQR	0.243	0.078	0.929	0.962	0.964
	AQR	0.776	0.656	0.917	0.955	0.957
	QGAM	0.776	0.148	**0.935**	0.966	0.967
Venda	PLAQR	0.939	0.994	0.914	0.960	0.956
	AQR	0.806	0.921	0.907	0.955	0.952
	QGAM	0.806	0.058	**0.923**	0.965	0.961

While the Ljung Box test provides a suitably robust alternative when the distribution of the response variable is heavily tailed, the BG test is the most appropriate residual serial autocorrelation test in the presence of a lagged response. Therefore, all of the models were valid to fit all data sets used for training. In addition, all coefficients of determination were at least $90\%$. That is, more than $90\%$ of the variations in the response were explained by the models. The very high R-square values indicate that all models learned the data very well and are very efficient in predicting solar irradiation. We note that QGAM had the highest R-square R-square values in bold) in all locations. The model explained variations in solar irradiation better than any of the models compared. Cross-validations results indicated that no model overfitted nor underfitted the data because the cross-validation correlations on the test data were all approximately equal to those on the training data (Table 4).

### Forecasting results

#### General model performances

All of Theil’s U statistics were less than one meaning that all models could fit the data better than corresponding naive models which could be fitted (Table 5). This means that all of the three non-parametric QR frameworks were suitable to model additive effects to SI. The QGAM model had the lowest AIC in all locations indicating that it fitted the data better than both the PLAQR and AQR models, though all of the AIC scores were approximately equal with regards to the locations. The RMSE values also confirm that the QGAM performed marginally the best in all locations because it had the lowest RMSE. However, the magnitudes of the RMSE scores were approximately the same.

**Table 5:** Model general performance metrics on the five different trained data.

Location	Model	Theil’s U	AIC	RMSE	MASE
Windhoek	PLAQR	0.185	96,991.8	94.717	0.137
	AQR	0.243	99,584.7	106.575	0.161
	QGAM	0.188	**97,543.4**	**90.757**	**0.123**
Alice	PLAQR	0.221	100,959.4	86.708	0.179
	AQR	0.228	102,968.2	92.608	0.201
	QGAM	0.196	**99,619.2**	**82.723**	**0.162**
Durban	PLAQR	0.200	100,208.6	84.241	0.181
	AQR	0.207	101,755.6	88.749	0.196
	QGAM	0.186	**100,028.7**	**81.622**	**0.170**
Pretoria	PLAQR	0.215	97,904.8	86.522	0.155
	AQR	0.248	100,302.3	94.059	0.177
	QGAM	0.210	**97,029.7**	**83.097**0	**0.142**
Venda	PLAQR	0.176	99,224.5	91.827	0.181
	AQR	0.188	100,831.2	95.892	0.195
	QGAM	0.182	**98,107.2**	**86.724**	**0.167**

Significant values are in bold.

**Figure 4:**
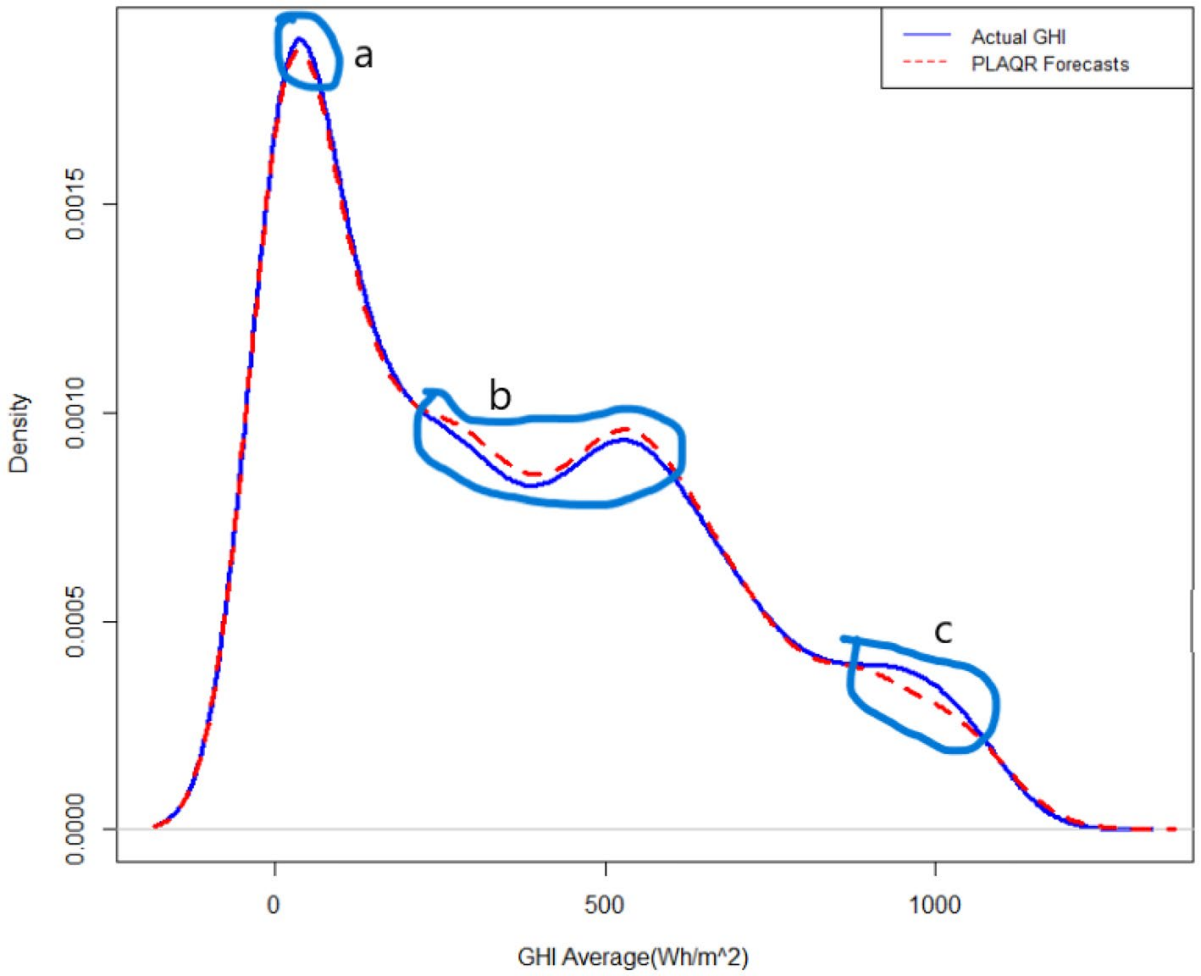
Forecasted density plot of GHI using the fitted PLAQR model.

**Figure 5:**
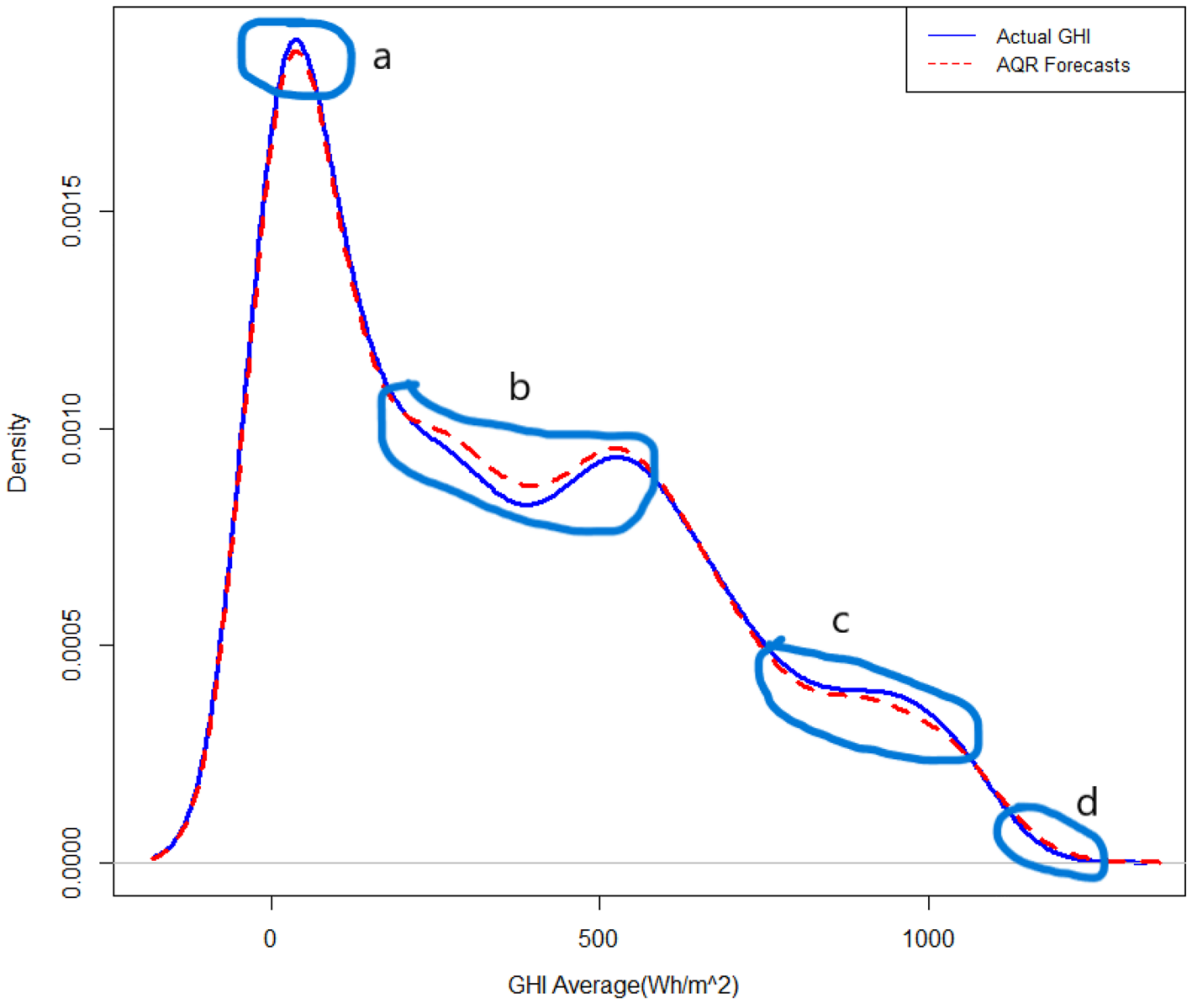
Forecasted density plot of GHI using the fitted AQR model.

**Figure 6:**
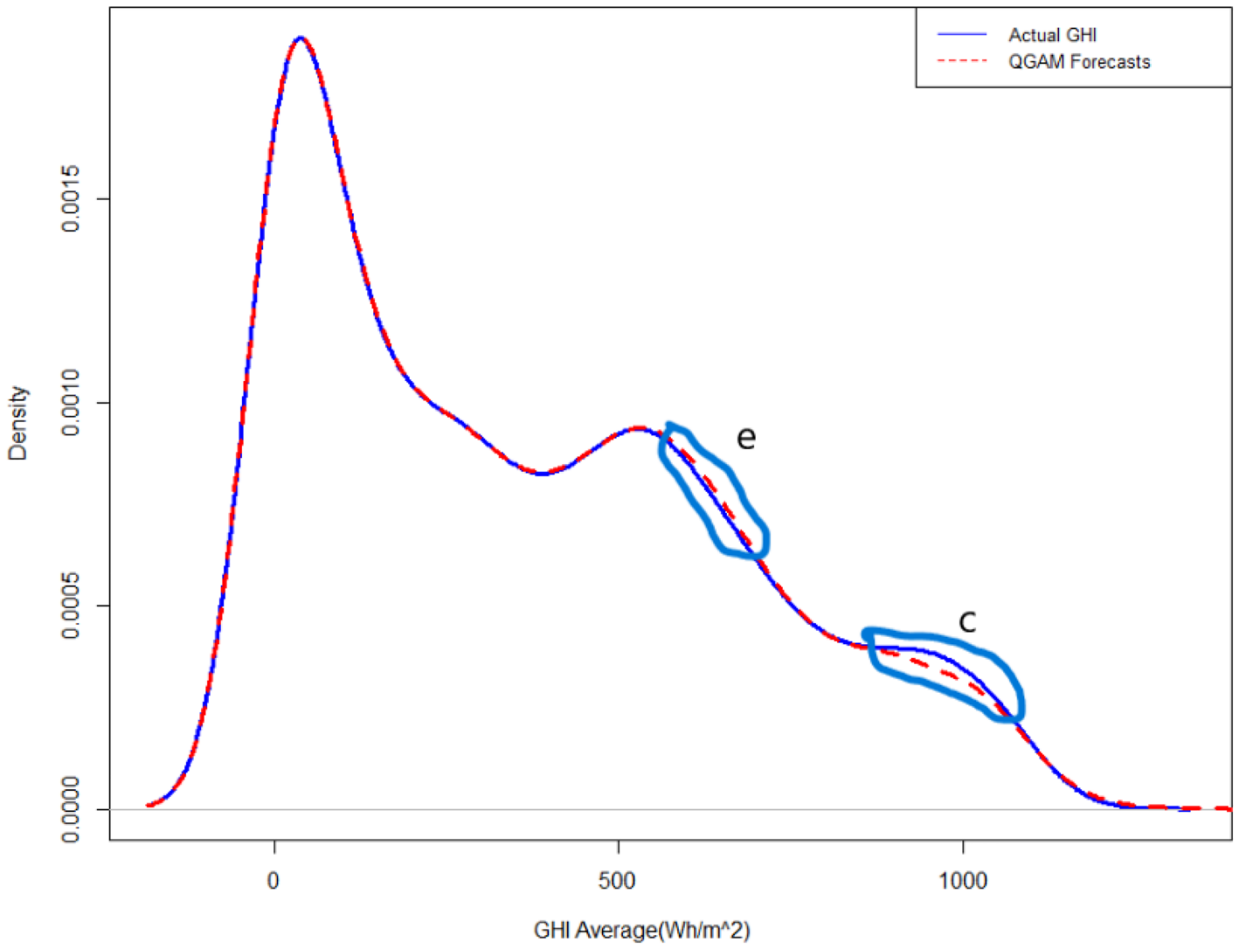
Forecasted density plot of GHI using the fitted QGAM model.

All mean absolute scaled error (MASE) scores were less than 1 meaning that all models performed better than a naïve benchmark. The MASE scores also demonstrate that QGAM predicted SI the most accurately by close margins because though the model had the lowest MASE in all locations the MASE scores were approximately equal. The MASE metric is one of the most appropriate metrics when the response has zero or near zero values like solar irradiation. The above three metric evaluations indicate that the three additive models have approximately the same out-of-sample forecasting performances. The forecasted density using the PLAQR is shown in Fig. 4. The model underestimated slightly the SI density in part (a) and notable in part (c) of the density plot. There is also a notable overestimation of the forecasted density in part (b). On the other hand, the AQR model did not estimate the forecasted density accurately on four different parts of the density plot. The model underestimated and overestimated the forecasted density on the same parts as the PLAQR model and additionally, slightly overestimated part (d) as shown in Fig. 5. Figure 6 for the QGAM exhibited the best-forecasted density because there are only two parts where the model did not estimate quite well the forecasted density. In the same part (c) as other models performed, the underestimation from QGAM was notable but slightly smaller than those from both the PLAQR and AQR models. However, the QGAM overestimated the forecasted density but on a different part (e) from the parts where the PLAQR and AQR models had overestimated. These results mean that the QGAM fitted the SI density a little closer to the actual density in all locations than the PLAQR and AQR models.

#### Sharpness and reliability analysis

**Figure 7:**
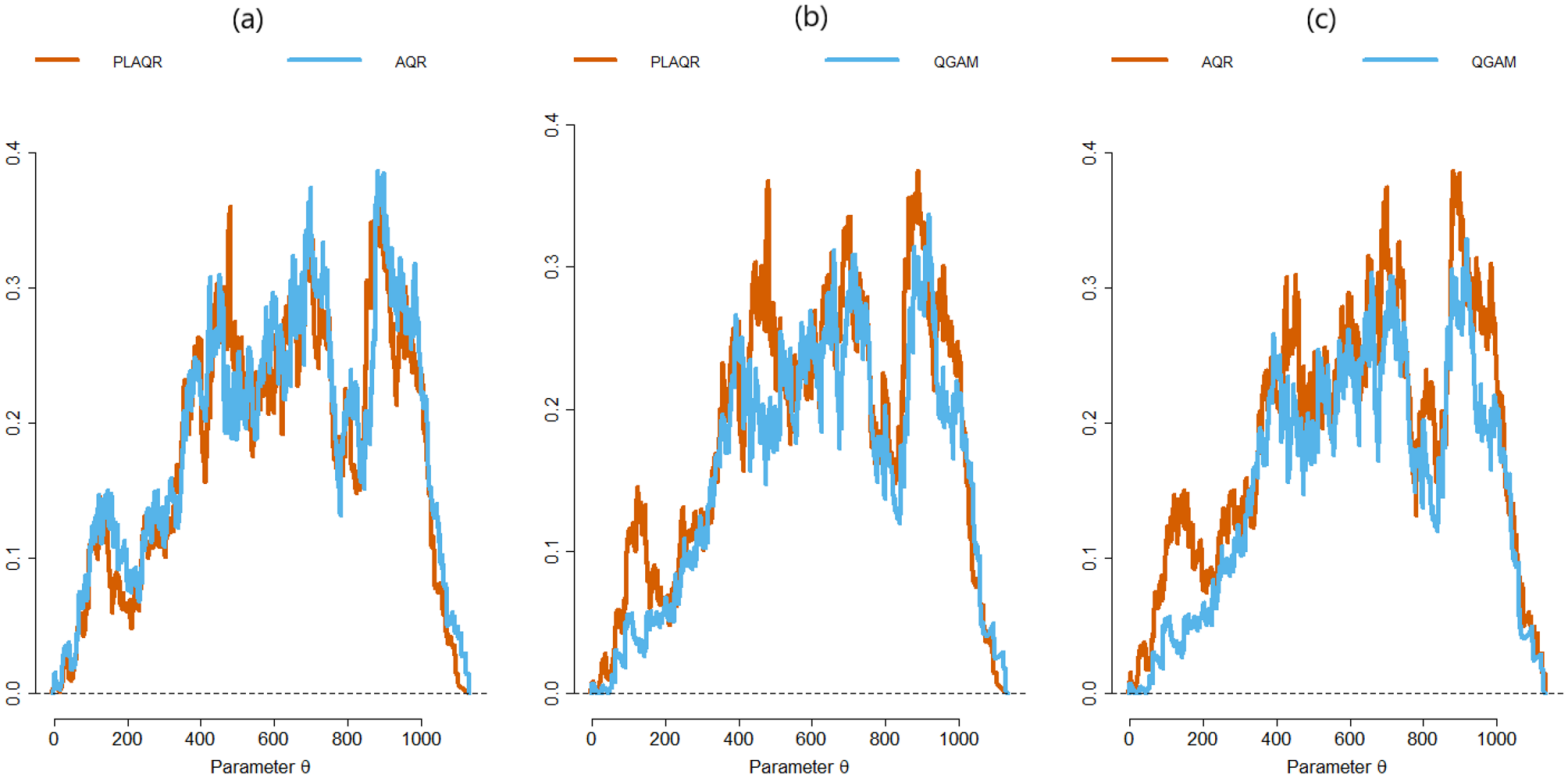
Murphy diagrams to compare the prediction accuracies of: (**a**) PLAQR and AQR models (**b**) PLAQR and QGAM models (**c**) AQR and QGAM models.

- *Metric Evaluations:* From Table 6 we can deduce that QGAM was the sharpest model and the most accurate on all locations because it had the lowest pinball loss in all locations. However, the pinball loss values from the QGAM were slightly smaller than those from the PLAQR and AQR models. We note that the pinball loss is an important metric when evaluating QR-based models. The lowest normalised Winkler scores were from the AQR model. Thus, AQR was the best model for the trade-off between coverage and prediction interval width but taking note of the slight differences in the normalised Winkler scores. The PLAQR was the most reliable except on Windhoek data because the model had the highest CP. However, results indicate that the CP values were slightly different and all models were reliable and unbiased because they had high CP values. The probabilistic metric evaluations demonstrate that the superiority in forecasting accuracy of the additive models depends on the metric but the models are generally of approximately the same forecasting accuracy.
- *Murphy Diagrams:* Murphy diagrams in Fig. 7 demonstrate that the QGAM had near best forecasts amongst the three quantile regression-based additive models though the curves were almost superimposed in many parts of the diagrams. The QGAM curve is slightly below that of PLAQR on the second Murphy diagram and also slightly below that of the AQR curve on the third Murphy diagram on some notable parts. In the first Murphy diagram, the curve for the AQR model is slightly above that of the PLAQR at low and high parametric values. From $\theta =400$ up to $\theta =800$ the PLAQR curve slightly is above that AQR. That is, the PLAQR model is more accurate than the AQR on $400\le \theta \le 800$. However, all of the Murphy diagrams had curves that were very close to each other. That is the QR-based additive models fitted had approximately the same accuracy at some degree of comparison.
- *Diebold–Mariano (DM) tests:* The DM tests were done on the covariate stationarity assumption which was validated in Section “Variable selection”. The following hypotheses: $H_0:$ *The PLAQR model has the same accuracy as the AQR model.* $H_1:$ *PLAQR model is less accurate than the AQR model.* were tested but all p-values in Table 7 were greater than 0.05 indicating that we could not reject the null hypothesis in all five locations. This means that the PLAQR and AQR models had generally the same accuracy. We also tested the hypotheses: $H_0:$ *PLAQR model has the same accuracy as the QGAM model.* $H_1:$ *PLAQR model is less accurate than the QGAM model.* and all p-values were less than 0.05 (Table 7) indicating that we could reject the null hypothesis. It then means that the accuracy of a PLAQR model is less than that of the QGAM model. The last pair of hypotheses tested were; $H_0:$ *AQR model has the same accuracy as the QGAM model,* $H_1:$ *AQR model is less accurate than the QGAM model,* and all of the p-values were less than 0.05 (Table 7) indicating that we could also reject the null hypothesis. That is, the accuracy of an AQR model is generally less than that of the QGAM model.
- *Performance consistency:* The forecasting performances of the models were checked separately for consistency through analysis of variance. The following assumptions were presumed valid without any loss of generality: (1) the performance scores were from random samples (random data sets used), (2) within each model set the performance scores were normal and (3) the mean performance may differ from one model to the other but the population standard deviation of the performance is the same for all models. That is, we analysed how the performances generally varied from one location to another using the following hypotheses: $H_0:$ *Model forecasting performance does not vary in all locations.* $H_1:$ *Model forecasting performance varies in at least one location.* The p-values obtained were all greater than 0.05 as shown in Table 8 indicating that we could not reject the null hypothesis. These results mean that we can conclude that all of the three models did not have varying forecasting performances across the locations. That is, they all had a consistent forecasting performance on solar irradiance. We can also conclude that the models were stable because location as a data variation factor did not influence the general performances of the three models.

**Table 6:** Model sharpness, biasedness, reliability and coverage metrics on the five different trained data.

Metric	Model	Windhoek	Alice	Durban	Pretoria	Venda
Pinball	PLAQR	25.37	26.20	24.93	24.84	26.75
Loss	AQR	29.90	29.43	27.03	28.36	28.87
	QGAM	**22.86**	**23.64**	**23.43**	**22.83**	**24.73**
	PLAQR	0.491	0.423	0.399	0.485	0.361
Winkler	AQR	**0.450**	**0.354**	**0.352**	**0.419**	**0.320**
	QGAM	0.514	0.464	0.427	0.494	0.383
	PLAQR	205.50	179.60	170.07	189.23	**179.47**
CRPS	AQR	**205.37**	**179.32**	**169.92**	189.24	179.50
	QGAM	205.39	179.61	170.15	**189.17**	179.52
	PLAQR	0.944	**0.946**	**0.945**	**0.940**	**0.949**
CP	AQR	0.885	0.926	0.891	0.900	0.927
	QGAM	**0.952**	0.935	0.934	0.935	0.940

Significant values are in bold.

**Table 7:** Diebold–Mariano tests p-values.

Location	PLAQRvsAQR	PLAQRvsQGAM	AQRvsQGAM
Windhoek	1.000	5.511E-14	1.889E-16
Alice	1.000	2.175E-10	2.200E-16
Durban	0.990	5.349E-05	4.196E-12
Pretoria	0.999	1.155E-13	2.241E-16
Venda	0.836	9.859E-07	5.599E-06

**Table 8:** ANOVA p-values on testing model consistency.

Model	F statistic	p-value
PLAQR	0.005	0.999
AQR	0.008	0.998
QGAM	0.006	1.000

#### Forecasting horizon effect

**Figure 8:**
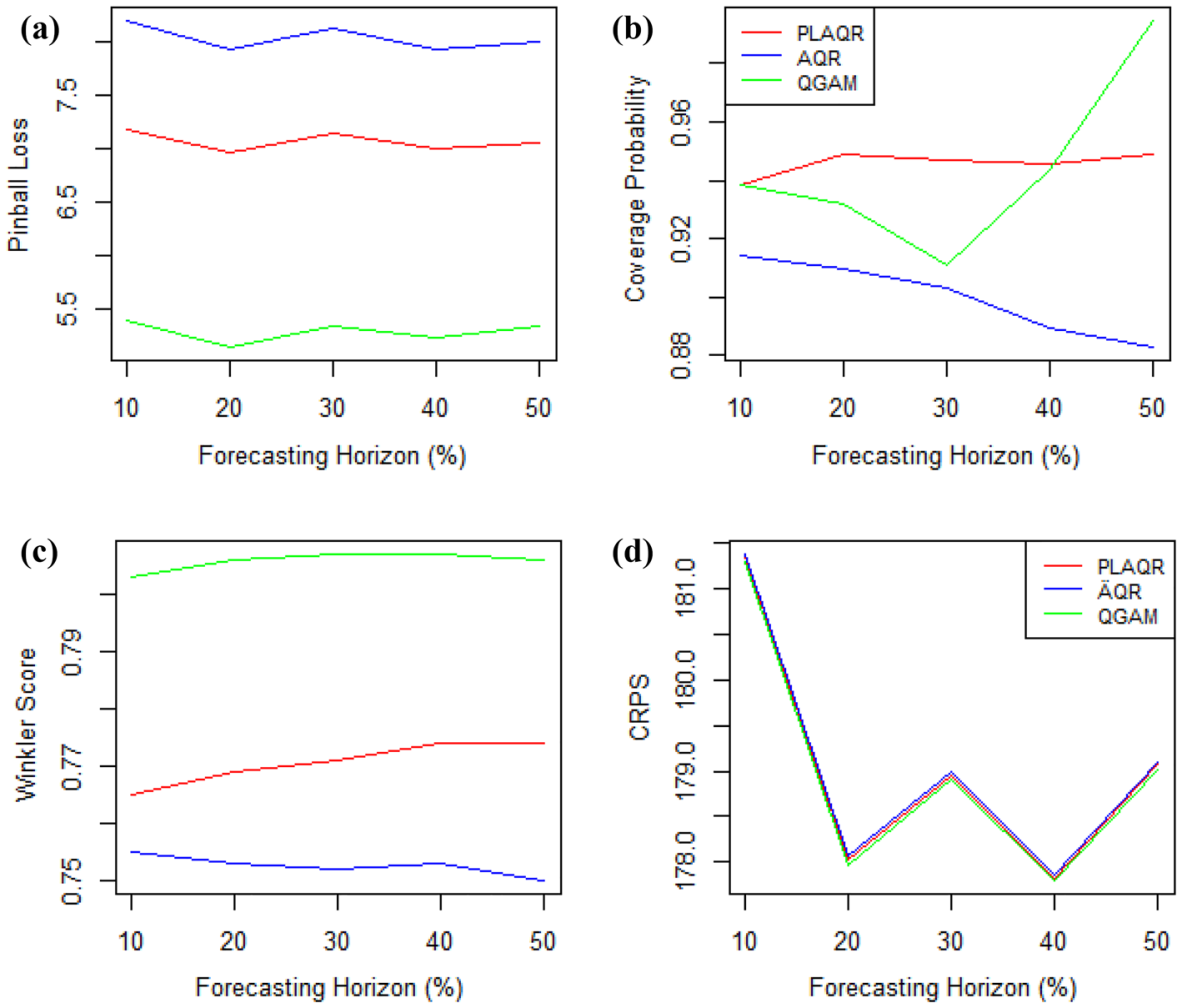
Forecasting horizon effect on model performance when considering (**a**) the pinball loss (**b**) CP (**c**) Winkler score (**d**) CRPS.

The sharpness of all models was not affected by the increase in the forecasting horizon and the QGAM has been the best overall forecasting horizon as shown in Fig. 8. Similarly, the trade-off between coverage and prediction interval width was not affected by the increase in forecasting horizon. However, the CP of the AQR model decreased with increasing forecasting horizon while that of QGAM had a turning point at $30\%$ forecasting horizon. In contrast, the CP of the PLAQR model was constant from $30\%$ throughout the increasing forecasting horizon. The models had approximately the same CRPS and results show that $20\%$ is the ideal horizon when forecasting the distribution.

#### Sample sife effect

**Figure 9:**
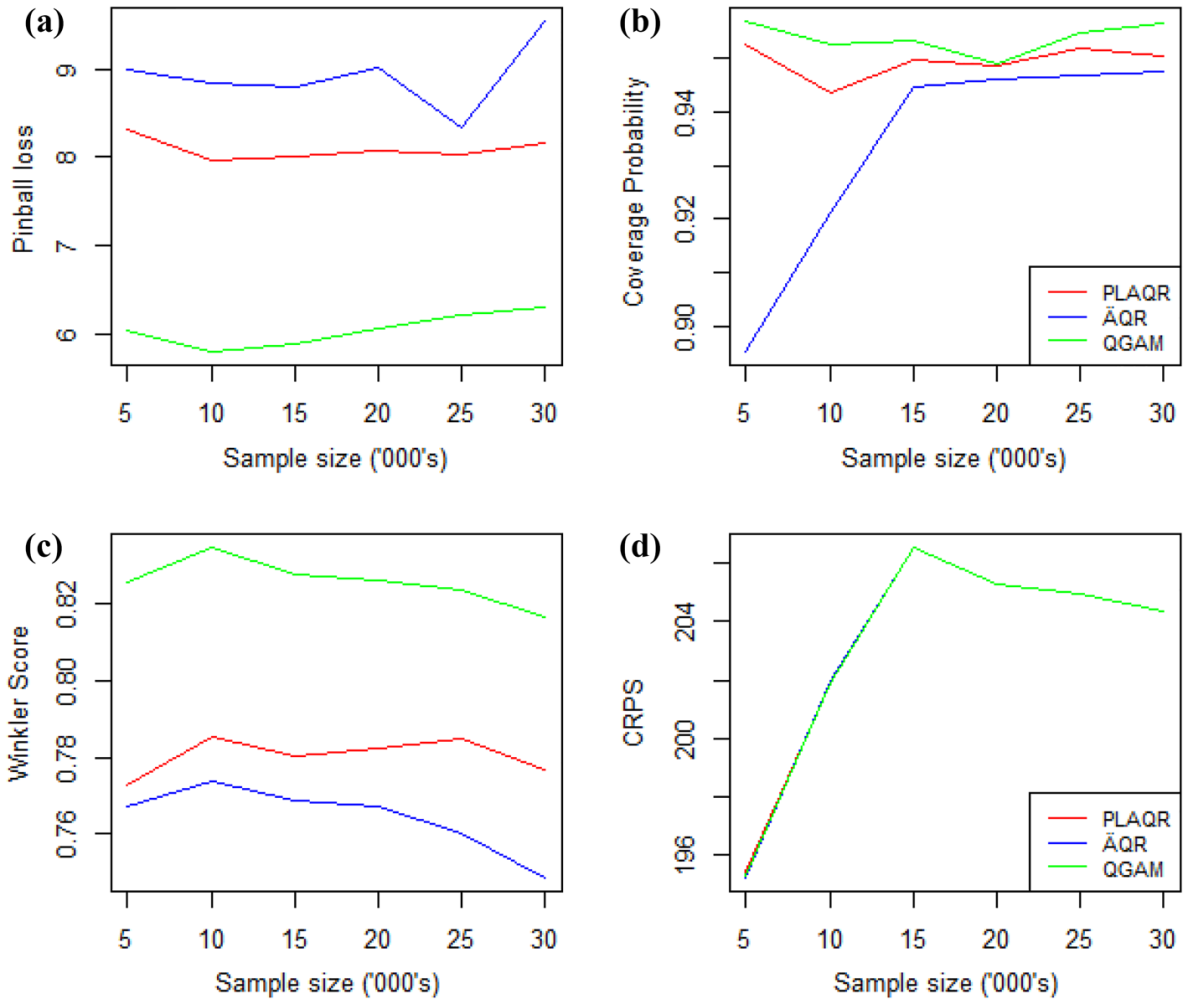
Sample size effect on model performance when considering (**a**) the pinball loss (**b**) CP (**c**) Winkler score (**d**) CRPS.

Model performances were not affected by changes in sample sizes as shown in Fig. 9 except the Winkler score. However, the movement from a sample size of 5000 to 10,000 influenced all models when considering the pinball loss, CP and Winkler scores evaluations. There is a general Winkler score improvement as the sample size increases while CP becomes approximately constant as the sample size increases from 15,000. We also note that the three models had the same CPRSs on all of the different sample sizes considered. Models’ performance on CRPS declines from the smallest sample size and then improves from the sample size of 15,000. Thus, 10,000 is a turning sample size for Pinball loss and Winkler score evaluation while 15,000 is the CRPS turning point.

## Discussions and conclusions

This study introduced the QGAM framework to forecasting SI using data from five different locations in Southern Africa. A comparative investigation against the PLAQR and AQR frameworks demonstrated their appropriateness in modelling additive effects. All three non-parametric additive frameworks based on quantile regression modelling fitted the data excellently and were highly valid to model SI data from the Southern Africa region. We attribute the excellent modelling capabilities, especially the very high coefficients of determination and cross-validation correlations to the models’ ability to avoid the curse of dimensionality while retaining great flexibility in the regression function^9^. In addition,^12^ concurred with^27^ that the use of B-splines makes additive models very stable and flexible for large-scale interpolation. The critical forecasting performance metric when fitting a QR-based model is the pinball loss. We think that the learning rate introduced by^21^ together with their replacement of the pinball loss with ELF loss function makes the QGAM framework very good and the best among the three models compared in minimising the regularised empirical risk suggested by^19^. The ELF loss function was developed as a smooth version of the pinball loss, so it led to slightly more accurate estimated quantiles. In as much as we suspected that some covariates have linear additive effects, the PLAQR framework which considers linear relationship structures was marginally outperformed by QGAM in all locations, forecasting horizons and different sample sizes except when evaluating the forecasts using the normalised Winkler score and CP. The PLAQR was the best model when evaluating the CP metric. The model uses a linear combination of B-spline basis functions to approximate the unknown nonlinear functions^8^. Probably that is why it had the highest coverage. However, all models were compared competitively very sharp, unbiased and very reliable because they had very high and approximately equal CP values. The QGAM performed the worst on the trade-off between coverage and PIW. The QGAM over- or under-estimated the SI density in fewer parts of the density plot than both the PLAQR and AQR models. Density plots of forecasts and actual GHI exhibit that QGAM predicted SI the closest. In addition, Murphy’s diagram analysis indicated that QGAM accuracy was slightly better than that of the other two non-parametric QR frameworks used to model the additive effects. Furthermore, the DM test results indicated that the QGAM framework had greater accuracy than both the PLAQR and AQR models. On the other hand, the DM test results indicated that the AQR model has a greater accuracy than the PLAQR model. We can deduce that smooth sub-optimisation of the EFL loss function within the maximum a posteriori estimation algorithm by exploiting orthogonal methods can account for the QGAM’s slightly greater accuracy than the other additive models. However, when prioritising reliability PLAQR is a recommended framework otherwise an AQR can be applied when focusing on the trade-off between coverage and PIW. The QGAM framework is recommended when focusing on the sharpness of the forecasts. Any of the three models can be used to predict the forecast distribution because they had approximately the same CRPS in all cases.

All of the models had different performances in the different locations but with no particular trend that could be established. That is, our results confirm the different model performances discovered by^18^ in different regions. Change of location elevation and grid coordinates did not have any effect on model performance. However, we note that all models performed the worst in Venda when evaluating the pinball loss. Results also show that the worst performance when using CRPS was from Windhoek otherwise it cannot be deduced where the models had the best performances. Therefore, we conclude that the change of location does not influence the forecasting performance of any modelling framework. We can attribute the change in model performance as we change locations to the qualities of the data sets from the different locations. By the way, data from different ground stations is recorded using different equipment and systems though it may be in similar formats.

This study also evaluated how the change in forecasting horizon may affect model performance. Results show that the pinball loss is not affected by the increase in forecasting horizon neither is the Winkler score. The CP and CRPS were the ones affected but differently on the three models. We can deduce that 30% is the turning forecasting horizon for all of the three models when measuring reliability. The performance of the models was approximately the same when measuring how accurately they forecasted the distribution throughout the increasing forecasting horizon. However, the zig-zag pattern exhibited is quite interesting and the CRPS improvement can be wildly deduced. We would wish to investigate what happens after the $50\%$ forecasting horizon but it is insensible to increase it beyond $50\%$. However, a forecasting horizon of $20\%$ is ideal.

At last, this study investigated how the increase in sample size affects model performance. It would seem that generally, the increase does not affect the pinball loss and CP but results show that a sample size of 10,000 is ideal for measuring the pinball loss and 15,000 on CP. The best Winkler score can be obtained from the largest possible sample size while increasing it from 15,000 does not affect the models’ reliability. Model performance was also approximately the same when measuring the CRPS throughout the increasing forecasting sample size. Another interesting discovery is that CRPS had a maximum sample size of 15,000. In contrast, smaller sample sizes had better CRPS. It can be concluded that 10,000 and 15,000 sample sizes are key when modelling additive effects to SI using non-parametric QR frameworks.

Though, the QGAM framework was marginally superior on six out of the ten metrics considered in this study, the models had approximately the same metric values. The approximately equal metric values computed, small differences in the densities forecasted and the same consistency and stability results can be attributed to the same B-splines structure used by all of the models to approximate non-parametric components. Thus, except for the DM test results, other comparison investigations in this study do not indicate outright superiority of the QGAM. It is also hihghlighted that incorporating a variety of evaluation metrics in forecasting analysis enhances the robustness, comprehensiveness, and relevance of performance assessment, ultimately leading to better-informed decisions and improvements in forecasting models. However, we recommend that a future simulation study can give more conclusive information on the comparative investigation between the non-parametric quantile regression models when modelling additive effects to SI. That is, our results can not be generalised to any other locational data sets except to those extracted from the same radiometric stations of the same localities until such a simulation study is done. However, a solar power generation system may prioritise at least one of the metrics among the pinball loss, Winkler score, CRPS and CP. Our results suggest a guideline on which forecasting framework to prioritise in such situations though all of the three additive models have demonstrated to have the same forecasting accuracies. The excellent forecasting performances and consistency exhibited by all of the 3 non-parametric QR models in this study entail that the frameworks should be highly regarded when a solar power system is predicting solar irradiance for their power generation planning and management. The results suggest that including additive models compared in this study in photovoltaic power generation can help stabilise the system through improved accurate SI forecasts. It has to be noted that this study can be extended to standardising forecasts and include forecast combinations in the discussed modelling frameworks to improve the forecasts. While the study focused on modelling additive effects, modelling frameworks like random forests can be introduced to the modelling of SI in future studies.

### Supplementary Information

Supplementary Information.

## Data Availability

Most of the data used in this study are from the SAURAN website (https://sauran.ac.za, accessed on 31 March 2023).
