# Supplementary material for: Non-parametric quantile regression-based modelling of additive effects to solar irradiation in Southern Africa
Source: Sci Rep. 2024 Apr 22;14:9244. doi: 10.1038/s41598-024-59751-8 (PMC11035626; doi:10.1038/s41598-024-59751-8)
Supplement: Supplementary file 1 — Supplementary Information. [file 41598_2024_59751_MOESM1_ESM.pdf]

Appendix A. Summary statistics

Descriptive statistics of covariates from each location.

Location	Variable	Min.	1st Qu.	Median	Mean	3rd Qu.	Max.
Windhoek	Hour	0.00	10.00	13.00	13.39	17.00	23.00
	RH	0.00	14.37	21.72	26.25	33.58	98.60
	Temp	1.835	20.740	24.630	24.347	28.770	36.810
	WS	0.034	1.313	1.948	2.008	2.625	6.513
	BP	799.0	834.0	836.0	835.8	838.0	845.0
	Lag1	0.1	158.2	501.9	490.1	772.2	1251.3
	Lag2	0.1	158.2	501.9	490.0	772.2	1251.3
Alice	Hour	0.0	9.0	13.0	12.5	16.0	23.0
	RH	6.406	38.520	55.410	56.320	74.457	100.000
	Temp	-1.256	15.533	19.970	20.149	24.727	42.650
	WD	0.308	116.500	166.400	171.148	228.700	360.000
	Lag1	0.1005	67.7716	301.0077	361.0563	581.5482	1154.6040
	Lag2	0.1005	67.6918	301.0077	361.0512	581.5482	1154.6040
Durban	Hour	0.00	9.00	12.00	12.31	16.00	23.00
	RH	19.39	69.79	78.42	77.16	86.70	100.00
	Temp	10.54	19.44	21.82	21.67	24.20	34.99
	WS	0.000	1.080	2.222	2.449	3.533	10.950
	WD	0.00	55.97	189.80	158.68	220.90	360.00
	Lag1	0.101	60.005	260.600	331.822	548.575	1141.000
	Lag2	0.101	59.987	260.600	331.782	548.575	1141.000
Pretoria	Hour	0	9	13	12.59	16	23
	RH	5.086	33.11	46.655	48.681	62.865	97.9
	Temp	2.205	17.692	21.325	21.237	25.050	36.020
	WS	0.005	1.128	1.835	2.072	2.852	8.080
	Lag1	0.1002	92.2048	321.9974	378.8982	632.6049	1179.1603
	Lag2	0.1002	92.2048	321.9974	378.8976	632.6049	1179.1603
Venda	Hour	0	9	12	12.39	16	23
	RH	5.487	41	56.46	57.408	4.665	100
	Temp	7.527	20.26	24.195	24.374	28.573	41.78
	Lag1	0.1002	92.2048	321.9974	378.8982	632.6049	1179.1603
	Lag2	0.1002	92.2048	321.9974	378.8976	632.6049	1179.1603
